# Human rickettsial pathogen modulates arthropod organic anion transporting polypeptide and tryptophan pathway for its survival in ticks

**DOI:** 10.1038/s41598-017-13559-x

**Published:** 2017-10-16

**Authors:** Vikas Taank, Shovan Dutta, Amrita Dasgupta, Tanner K. Steeves, Durland Fish, John F. Anderson, Hameeda Sultana, Girish Neelakanta

**Affiliations:** 1 Department of Biological Sciences, Old Dominion University, Norfolk, VA USA; 2 School of Public Health, Yale University School of Medicine, New Haven, CT USA; 3 Department of Entomology, Connecticut Agricultural Experiment Station, New Haven, CT USA; 4 Center for Molecular Medicine, Old Dominion University, Norfolk, VA USA; 5 Present Address: Skin of Color Research Institute, Hampton University, Hampton, VA USA

## Abstract

The black-legged tick *Ixodes scapularis* transmits the human anaplasmosis agent, *Anaplasma phagocytophilum*. In this study, we show that *A. phagocytophilum* specifically up-regulates *I. scapularis* organic anion transporting polypeptide, *isoatp4056* and kynurenine amino transferase (*kat*), a gene involved in the production of tryptophan metabolite xanthurenic acid (XA), for its survival in ticks. RNAi analysis revealed that knockdown of *isoatp4056* expression had no effect on *A. phagocytophilum* acquisition from the murine host but affected the bacterial survival in tick cells. Knockdown of the expression of *kat* mRNA alone or in combination with *isoatp4056* mRNA significantly affected *A. phagocytophilum* survival and *isoatp4056* expression in tick cells. Exogenous addition of XA induces *isoatp4056* expression and *A. phagocytophilum* burden in both tick salivary glands and tick cells. Electrophoretic mobility shift assays provide further evidence that *A. phagocytophilum* and XA influences *isoatp4056* expression. Collectively, this study provides important novel information in understanding the interplay between molecular pathways manipulated by a rickettsial pathogen to survive in its arthropod vector.

## Introduction

Human anaplasmosis caused by an obligate intracellular pathogen, *Anaplasma phagocytophilum*, is one of the most common arthropod-borne diseases in the United States, Europe and Asia^1–3^. *A. phagocytophilum* has developed various strategies for its survival and infection of diverse hosts that include humans, horses, cattle, deer, sheep, mice and reindeer among other species^4–8^. Various genetic variants of this bacterium have been identified and characterized in different animals^9–12^. In northeastern United States, the blacklegged tick, *Ixodes scapularis*, serve as the primary vector for this pathogen. These ticks acquire *A. phagocytophilum* upon feeding on an infected animal^3,13^. Upon entry through blood feeding, *A. phagocytophilum* first enters into the midgut and then establishes itself in the salivary glands of these ticks^13^. Transovarial transmission does not occur. However, *A. phagocytophilum* can be transstadially transmitted to different arthropod developmental stages^13^. The survival strategies that this bacterium uses to persist in different arthropod developmental stages, particularly in unfed stage, are not clearly understood. Understanding survival strategies of *A. phagocytophilum* in arthropod unfed stages would reveal important insights on the mechanism(s) on how this bacterium could persist during tick molting phases.

In humans, *A. phagocytophilum* primarily infects and persists within human neutrophils by delaying apoptosis, inhibiting NADPH oxidase activity and subverting phagolysosome biogenesis^11,12^. In addition, *A. phagocytophilum* injects some of the type IV effector proteins to modulate mammalian host cell signaling^11,12^. While much is known about the interactions of *A. phagocytophilum* with mammalian cells, very little is known about how *A. phagocytophilum* interacts and survives within the arthropod vector. *A. phagocytophilum* specifically induces and represses several of the tick genes^14–19^. Several studies have used *in vitro* tick cells to understand interactions of *A. phagocytophilum* ^20–26^. Our previous efforts were focused on the use of both tick cells and murine models to understand the dynamics of *A. phagocytophilum*-tick interactions^16,19^. Even though some studies have identified novel tick molecules in the interactions with this bacterium, the detailed molecular mechanisms of the importance of tick molecules in *A. phagocytophilum* survival in these ticks remains to be elucidated.

Human organic anion and cation transporters are classified into organic anion transporting polypeptides (OATPs), organic anion transporters (OATs) and organic cation transporters (OCTs)^27^. These families of proteins are highly conserved molecules among various vertebrates that play important roles in the influx of several metabolites including xanthurenic acid (XA), a metabolite from the tryptophan oxidation pathway^27–30^. Humans encode 11 OATPs that are localized to barrier epithelial cells^27,29^. These OATPs are involved in uptake and transcellular movement of several substrates such as signaling molecules, hormones, growth factors, toxins and xenobiotics across body fluid compartments^29,31–33^. Recent studies on human OATPs have emphasized their role in inter-organ communication between brain, eye, liver and kidneys^27,29,31–33^. Topology predictions of human OATPs suggest that two pairs of 6-transmembrane domains are connected by a large intracellular loop with several potential posttranslational modifications^27,31–33^. Both N-terminus and C-terminus portions of OATPs are predicted to be intracellular^27,31,32^.

Recent studies have reported that OATPs are also conserved among various arthropod species^34,35^. *I. scapularis* ticks encode nine OATPs that are expressed in various tissues, including salivary glands^35^. Some of these OATPs are shown to be important for blood feeding by ticks^34^. The knowledge on the role of this important class of molecules in vector-pathogen interactions is lacking. Using *A. phagocytophilum-I. scapularis* as a model, we provide evidence to show that tick-borne pathogens modulate these highly conserved molecules and the tryptophan metabolism pathway for its survival in the medically important arthropod vector.

## Results

### *A. phagocytophilum* induces expression of specific *I. scapularis* organic anion transporting polypeptides (OATPs) in unfed ticks

**Figure 1:**
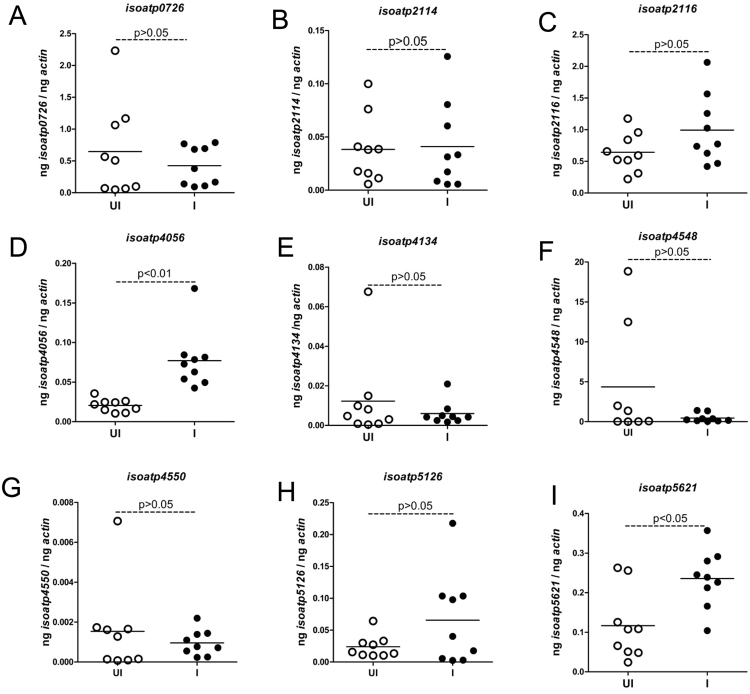
*A. phagocytophilum* up-regulates *isoatp4056* and *isoatp5621* in unfed *I. scapularis* nymphal ticks. (**A**–**I**) Quantitative PCR analysis showing expression of nine OATPs: *isoatps*-*0726* (**A**), -*2114* (**B**), -*2116* (**C**), -*4056* (**D**), -*4134* (**E**), -*4548* (**F**), -*4550* (**G**), -5126 (**H**) and -5621 (**I**) between unfed uninfected ticks and *A. phagocytophilum*-infected ticks. Open circle represents uninfected (UI) and closed circles represent infected (**I**) ticks. Each circle represents one tick. The mRNA levels of OATPs are normalized to tick beta-actin mRNA levels. P value from non-paired Student’s t-test is shown.

*I. scapularis* ticks encode nine OATPs that are highly expressed in unfed ticks^35^. The influence of tick-borne pathogens on the expression of OATPs is currently not explored. Therefore, expression of all nine OATPs was determined in unfed *I. scapularis* nymphal ticks infected with *A. phagocytophilum* (Fig. 1). Unfed *A. phagocytophilum*-infected nymphal ticks were generated as described in methods. QRT-PCR analysis revealed no significant differences in the expression levels of *isoatps*-0726 (Fig. 1A), -2114 (Fig. 1B), -2116 (Fig. 1C), -4134 (Fig. 1E), -4548 (Fig. 1F), -4550 (Fig. 1G), and -5126 (Fig. 1H) between unfed uninfected ticks and *A. phagocytophilum*-infected ticks. However, expression of *isoatp4056* (Fig. 1D) and *isoatp5621* (Fig. 1I) was significantly (P < 0.05) upregulated in the presence of *A. phagocytophilum* in comparison to the uninfected controls. These results show that *A. phagocytophilum* induces expression of specific tick OATPs (*isoatp4056* and *isoatp5621*) in unfed ticks.

### Expression of both *I. scapularis isoatp4056* and *isoatp5621* are developmentally regulated but only *isoatp4056* is induced upon *A. phagocytophilum* colonization in the tick salivary glands

**Figure 2:**
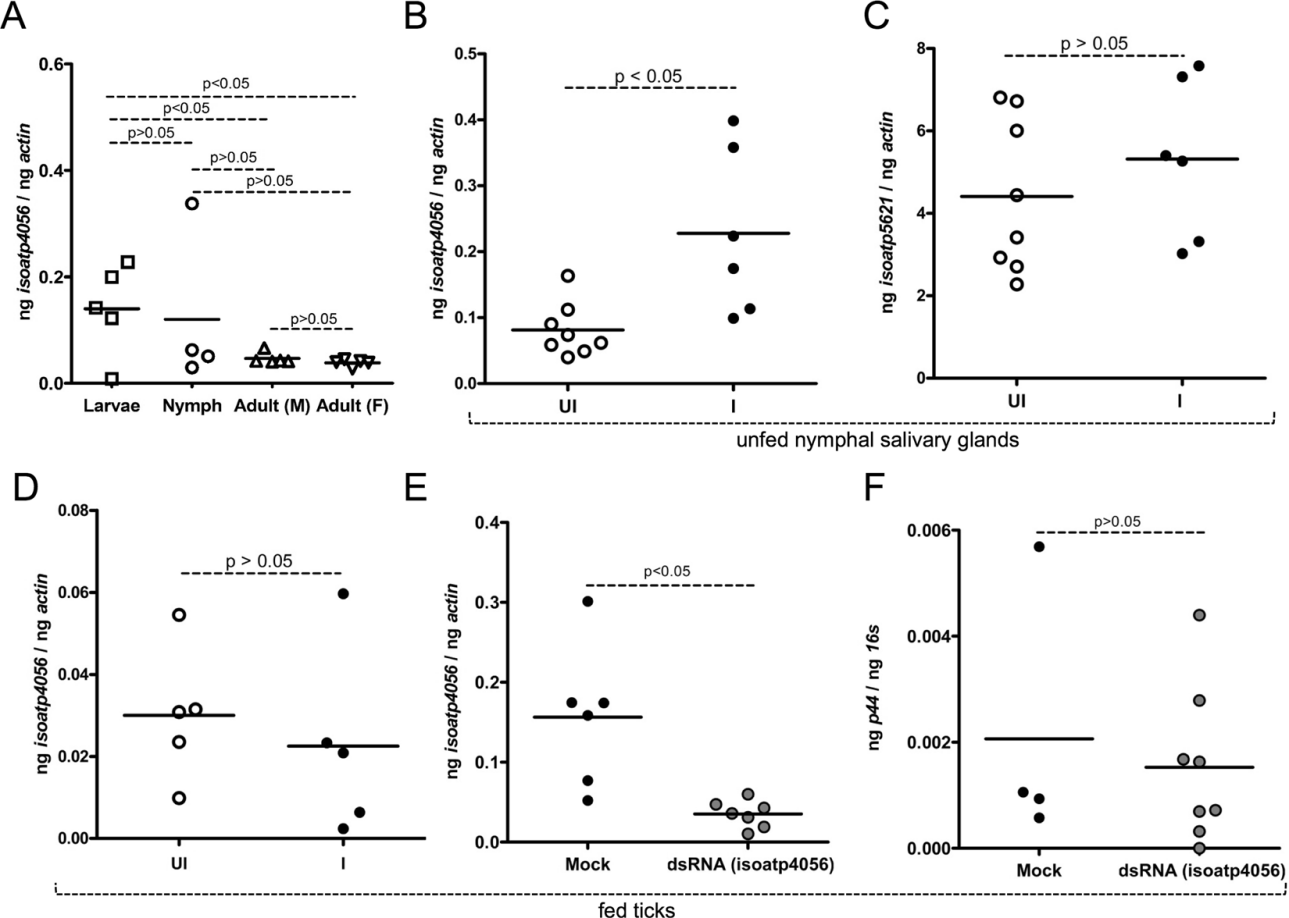
*Isoatp4056* is upregulated in unfed *A. phagocytophilum*-infected *I. scapularis* nymphal salivary glands and has no role in bacterial acquisition from murine host to ticks. Quantitative PCR analysis showing expression of *isoatp4056* at different tick developmental stages in uninfected unfed ticks (**A**) and in the nymphal salivary glands of uninfected (UI) and infected (I) unfed nymphs (**B**) is shown. In panel A, data for larvae samples was obtained from 5–7 pooled ticks and for nymphs, adult male (M) and female (F) ticks each circle or triangle indicates individual tick. (**C**) QRT-PCR analysis of *isoatp5621* transcripts in unfed nymphal salivary glands is shown. (**D**) QRT-PCR analysis showing expression of *isoatp4056* in ticks during acquisition in uninfected and *A. phagocytophilum*-infected nymphal ticks. (**E**) The *isoatp4056* mRNA levels in ticks treated with mock or *isoatp4056*-dsRNA during acquisition is shown. (**F**) *A. phagocytophilum* burden in mock or dsRNA-treated ticks during acquisition (48 h post repletion) is shown. The mRNA levels of OATPs are normalized to tick beta-actin mRNA levels and levels of P44 (*A. phagocytophilum* burden) were normalized to tick 16S levels. Open circle represents uninfected (UI) and closed circles represent infected (I) ticks in B, C and D. Close circle represents infected-mock treated and gray circle represents infected-*isoatp4056*-dsRNA- treated ticks. Each circle represents one tick. P value from non-paired Student’s t-test is shown.

We then determined whether expression of *isoatp4056* and *isoatp5621* was regulated during tick developmental stages. The *isoatp4056* and *isoatp5621* mRNA levels were assessed by QRT-PCR in unfed uninfected ticks at different tick developmental life stages using tick beta-actin as control (Fig. 2A and Supplementary Fig. 1). The *isoatp4056* gene was expressed in all tick developmental stages, where, larvae expressed significantly (P < 0.05) higher levels in comparison to both male and female adult ticks (Fig. 2A). No significant differences (P > 0.05) in the *isoatp4056* mRNA levels were seen between larvae and nymphs or between nymphs and adult ticks (Fig. 2A). Comparison of *isoatp4056* expression between adult males and female ticks revealed no differences (Fig. 2A). The *isoatp5621* expression was evident in all the developmental life stages of ticks, where, nymphs expressed significantly (P < 0.05) higher levels in comparison to both male and female adult ticks (Supplementary Fig. 1A). No significant difference (P > 0.05) in *isoatp5621* expression was evident between larvae and nymphs or between larvae and adult ticks or between male and female adult ticks (Supplementary Fig. 1A). Furthermore, we observed that the expression of *isoatp4056* transcripts was significantly (P < 0.05) elevated upon *A. phagocytophilum* colonization in unfed nymphal salivary glands (Fig. 2B). In contrast, no difference in the levels of *isoatp5621* transcripts in salivary glands was observed between uninfected and *A. phagocytophilum*-infected ticks (Fig. 2C). In addition, no significant difference was evident neither in *isoatp4056* (Supplementary Fig. 1B) nor in *isoatp5621* (Supplementary Fig. 1C) expression in unfed nymphal guts isolated from uninfected or *A. phagocytophilum-infected* ticks. These results show that *A. phagocytophilum* specifically regulate expression of *isoatp4056* but not *isoatp5621* in tick salivary glands.

### RNA interference (RNAi) of *isoatp4056* expression in ticks has no effect on the *A. phagocytophilum* acquisition from the murine host

We then determined levels of *isoatp4056* transcripts during acquisition of *A. phagocytophilum* into naïve nymphal ticks from the infected murine host. The levels of *isoatp4056* transcripts were unaltered in the presence of *A. phagocytophilum* during acquisition into naïve nymphal ticks from the murine host in comparison to the uninfected controls (Fig. 2D). To further analyze whether IsOATP4056 has any role in the acquisition of *A. phagocytophilum* from infected murine host, we generated *isoatp4056*-deficient unfed naïve nymphal ticks by RNA interference (RNAi). Equal volumes containing *isoatp4056*-dsRNA or mock control were microinjected into the body of unfed naïve nymphal ticks. Microinjected ticks were allowed to re-acclimatize for 2–3 hours at room temperature and immediately fed on *A. phagocytophilum*-infected mice. QRT-PCR analysis revealed a significant reduction of *isoatp4056* mRNA in the *isoatp4056*-dsRNA-injected ticks compared with the mock-injected controls (Fig. 2E). However, no differences in the levels of *A. phagocytophilum* were seen between mock or *isoatp4056*-dsRNA injected nymphal ticks (Fig. 2F). These results suggest that *isoatp4056* has no role in the acquisition of *A. phagocytophilum* from the murine host to ticks.

### RNAi of *isoatp4056* expression affects *A. phagocytophilum* survival in tick cells

**Figure 3:**
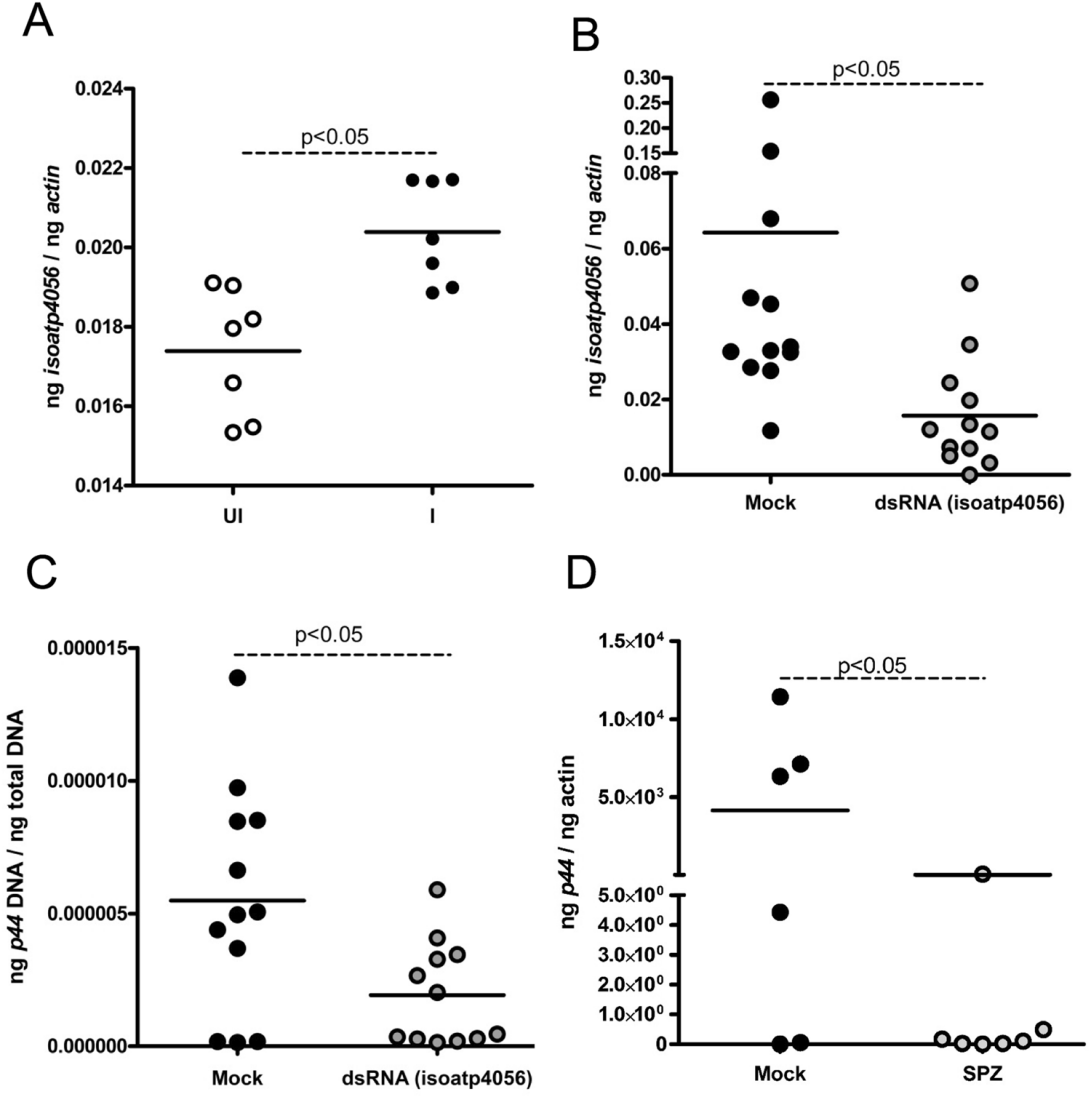
RNAi-mediated silencing of *isoatp4056* in tick cells affects *A. phagocytophilum* growth. (**A**) QRT-PCR analysis showing expression of *isoatp4056* upon *A. phagocytophilum* infection (**A**) or upon treatment with mock or *isoatp4056*-dsRNA (**B**) in tick cells is shown. UI indicates uninfected and I indicate *A. phagocytophilum*-infected tick cells. (**C**) *A. phagocytophilum* burden in mock or *isoatp4056*-dsRNA-treated tick cells is shown. The data in (**B**) and (**C**) are from *A. phagocytophilum*-infected ISE6 tick cells treated with mock or *isoatp4056*-dsRNA. Each circle represents data from one independent culture plate well. The mRNA levels of *isoatp4056* are normalized to tick beta-actin levels and the levels of P44 (*A. phagocytophilum* burden) were normalized to tick total DNA levels. (**D**) *A. phagocytophilum* burden in mock or 100 μM of OATP inhibitor ± -sulfinpyrazone-treated (SPZ) tick cells is shown. P value from non-paired Student’s t-test is shown.

We then tested the effect of *isoatp4056-*silencing on the bacterial survival in tick cells. The levels of *isoatp4056* transcripts were significantly (P < 0.05) upregulated in tick cells upon *A. phagocytophilum* infection in comparison to the uninfected controls (Fig. 3A). We then addressed whether silencing of *isoatp4056* in tick cells impact *A. phagocytophilum* survival. Transfection analysis with *isoatp4056*-dsRNA in the uninfected ISE6 cells did not reveal any change on cell morphology at 4 and 24 h post-transfection in comparison to mock-treated cells (Supplementary Fig. 2A) but clearly showed a significant (P < 0.05) reduction of *isoatp4056* transcripts in *isoatp4056*-dsRNA-treated cells (Supplementary Fig. 2B). The QRT-PCR analysis also revealed significant (P < 0.05) reduction of *isoatp4056* mRNA in the *A. phagocytophilum-*infected *isoatp4056*-dsRNA-treated tick cells in comparison to the mock-treated controls (Fig. 3B). In addition, no visual morphological changes were evident in the *A. phagocytophilum*-infected (24 p.i.) tick cells treated with either *isoatp4056*-dsRNA (48 h post-treatment) in comparison to mock control (Supplementary Fig. 2A). However, RNAi-mediated silencing of *isoatp4056* expression in *A. phagocytophilum*-infected-*isoatp4056*-dsRNA-treated tick cells showed significant (P < 0.05) reduction in the bacterial burden in comparison to the *A. phagocytophilum*-infected-mock-treated controls (Fig. 3C). To further support the RNAi analysis, we tested the effect of commercially available OATP inhibitor (± -sulfinpyrazone) on *A. phagocytophilum* infection of tick cells. Tick cells were treated with 100 μM of ± -sulfinpyrazone followed by *A. phagocytophilum* infection as described in the methods section. QRT-PCR analysis revealed that *A. phagocytophilum* burden was significantly (P < 0.05) decreased in tick cells upon treatment with 100 μM of ± -sulfinpyrazone (Fig. 3D) in comparison to the mock-treated control. Collectively, these results suggest that IsOATP4056 play a role in the survival of *A. phagocytophilum* in tick cells.

### *A. phagocytophilum* up-regulates kynurenine aminotransferase *(kat)*, a gene involved in the synthesis of xanthurenic acid (XA), in the salivary glands of unfed ticks

**Figure 4:**
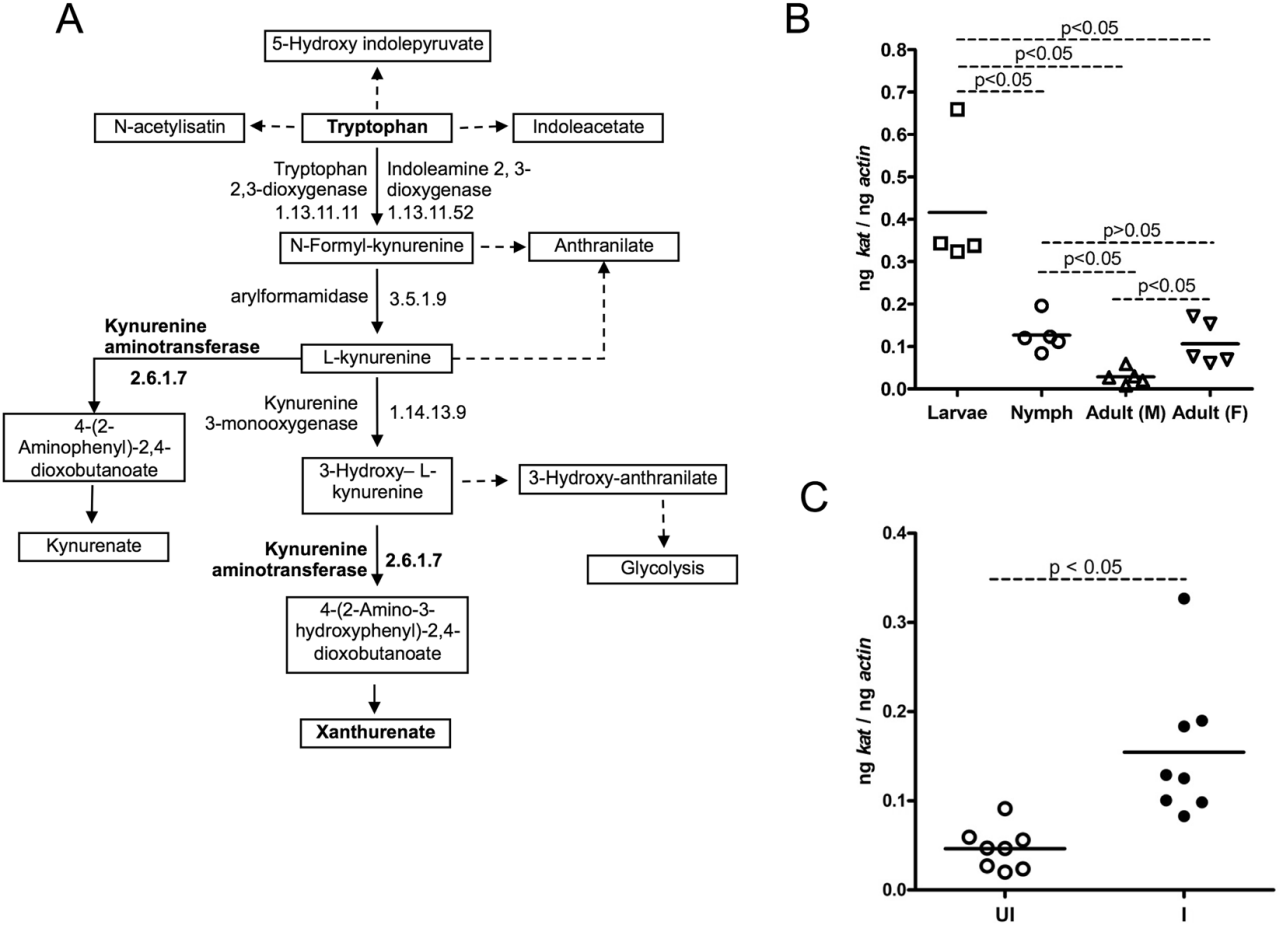
*A. phagocytophilum* up-regulates kynurenine aminotransferase (*kat*) gene in unfed *I. scapularis* salivary glands. (**A**) Putative tryptophan metabolism pathway in *I. scapularis* ticks is shown. Tryptophan pathway was modified to simple format from KEGG pathway: isc00380 for the purpose of this study. Xanthurenate and kynurenate are some of the products from this pathway. Kynurenine aminotransferase, Xanthurenate and Tryptophan are shown in "bold" text. Quantitative PCR analysis showing expression of *kat* in different developmental stages in uninfected unfed ticks (**B**) or in the salivary glands (**C**) of uninfected (UI) and *A. phagocytophilum*-infected (I) unfed nymphs is shown. Data for larvae samples was obtained from 5–7 pooled ticks. Open circle represents uninfected (UI) and closed circles represents infected (I) ticks. Each circle represents one tick. Expression of *kat* mRNA was normalized to tick beta-actin. P value from non-paired Student’s t-test is shown.

The upregulation of *isoatp4056* transcripts in the salivary glands of unfed ticks and in tick cells suggests its important role in the survival and colonization of *A. phagocytophilum* in tick cells. Studies have shown that human OATs (structurally similar to OATPs) are the transporters for XA^30^. Ticks encode kynurenine aminotransferase (KAT) that could participate in the synthesis of both kynurenate and xanthurenate (Fig. 4A, Supplementary Table 1). *I. scapularis* KAT (GenBank acc. no. XM_002401267) shows 70.8% identity with *Haemaphysalis longicornis* HIKAT, for which catalysis of transamination of 3-hydroxykynurenine to XA is reported^36^. Several other genes involved in tryptophan metabolism are also present in *I. scapularis* genome (Supplementary Table 1). QRT-PCR analysis revealed developmental regulation of *kat* transcripts with significant (P < 0.05) increased levels in the larval ticks in comparison to the other developmental stages of ticks (Fig. 4B). We also noticed significantly (P < 0.05) increased *kat* mRNA levels in the unfed nymphal ticks in comparison to unfed adult male ticks and in unfed adult female ticks in comparison to the unfed adult male ticks (Fig. 4B). Increased levels of *kat* mRNA were also evident in the tick salivary glands of unfed ticks in the presence of *A. phagocytophilum* in comparison to uninfected controls (Fig. 4C). These results suggest a role for KAT in the interactions with *A. phagocytophilum* during its colonization in the unfed ticks.

### RNAi of *kat* expression affects *A. phagocytophilum* burden and expression of *isoatp4056* in tick cells

**Figure 5:**
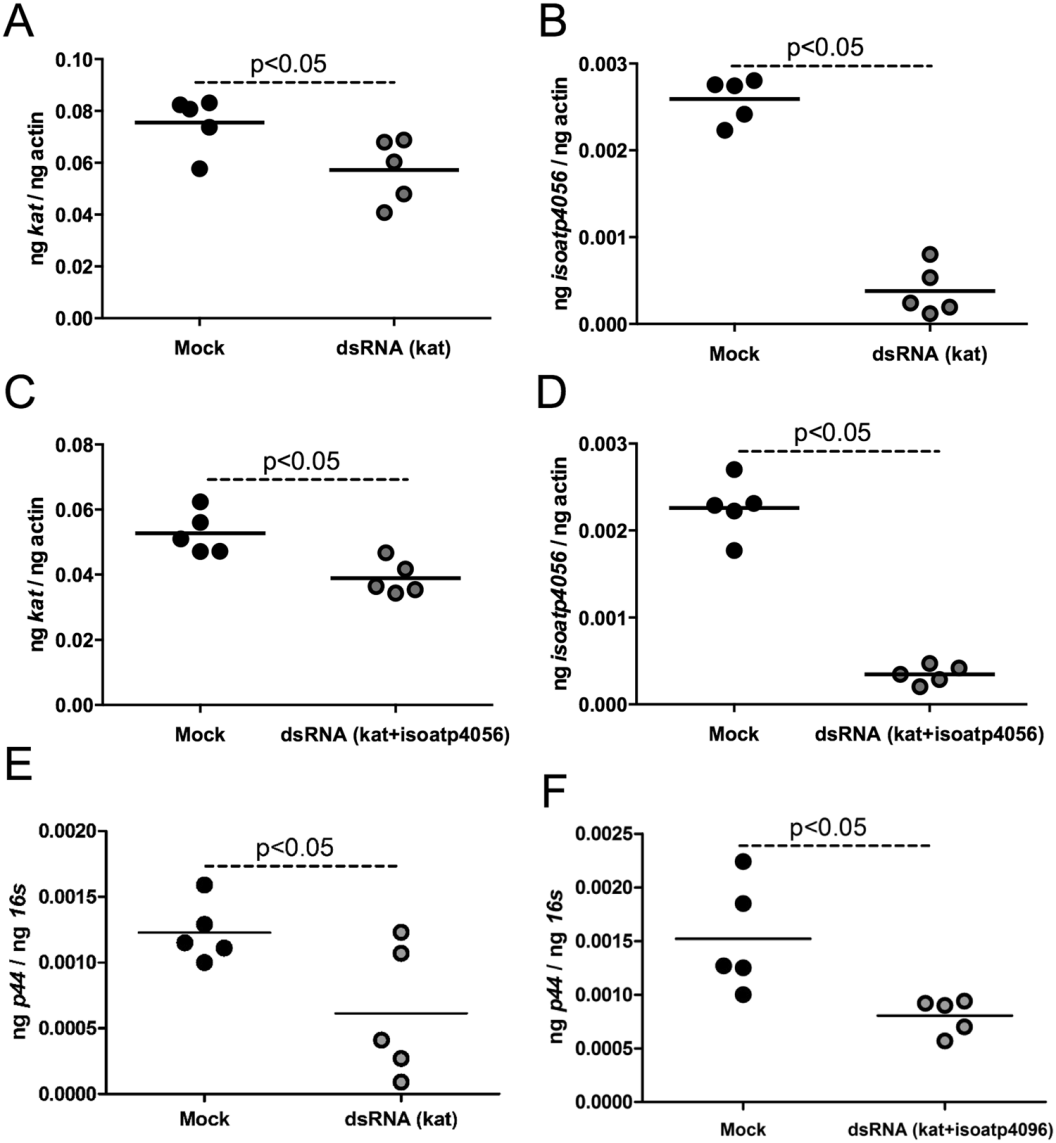
RNAi-mediated silencing of *kat* in tick cells affects *A. phagocytophilum* growth and *isoatp4056* expression. QRT-PCR analysis showing expression of *kat* (**A** and **C**) or *isoatp4056* (**B** and **D**) in *A. phagocytophilum-*infected mock or *kat*- (**A** and **B**) or *kat* + *isoatp4056*-dsRNA-treated (**C** and **D**) tick cells is shown. *A. phagocytophilum* burden in mock or *kat*- (**E**) or *kat* + *isoatp4056*-dsRNA-treated (**F**) tick cells is shown. Each circle represents data from one independent culture plate well. Black circles indicate data from mock-treated and gray circles represents data from either *kat*-dsRNA-treated or *kat* + *isoatp4056*-dsRNA-treated tick cells. The mRNA levels of *kat* or *isoatp4056* are normalized to tick beta-actin levels and the levels of P44 (*A. phagocytophilum* burden) were normalized to tick 16S levels. P value from non-paired Student’s t-test is shown.

As *kat* transcripts were upregulated upon *A. phagocytophilum* infection, we tested the effect of *kat-*silencing on the bacterial survival in tick cells. Transfection analysis in tick cells did not reveal any morphological differences between mock-or *kat*-dsRNA-treated cells neither at 4 and 24 h post-transfection (Supplementary Fig. 3A) nor at 24 h p.i. (48 h post-transfection). The QRT-PCR analysis revealed significant (P < 0.05) reduction of *kat* mRNA in the *A. phagocytophilum-*infected *kat*-dsRNA-treated tick cells in comparison to the mock-treated controls (Fig. 5A). In addition, significant (P < 0.05) reduction in *isoatp4056* mRNA levels was noted upon silencing of *kat* gene expression in these cells (Fig. 5B). Simultaneous silencing of both *kat* and *isoatp4056* transcripts had no significant morphological changes in *A. phagocytophilum*-infected ticks cells in comparison to infected mock-treated cells (Supplementary Fig. 3B). However, significantly (P < 0.05) reduced levels of both *kat* (Fig. 5C) and *isoatp4056* (Fig. 5D) mRNA was evident in *A. phagocytophilum*-infected tick cells treated with both *kat*- and *isoatp4056-*dsRNA in comparison to mock-treated control. Furthermore, QRT-PCR analysis revealed significant (P < 0.05) reduction of bacterial burden in both groups of *A. phagocytophilum*-infected tick cells treated with *kat*-dsRNA alone (Fig. 5E) or in combination with *isoatp4056*-dsRNA (Fig. 5F). Taken together, these results suggest that the interplay between KAT and IsOATP4056 pathways is critical for *A. phagocytophilum* survival and colonization in tick cells.

### Exogenous addition of XA induces *isoatp4056* expression and *A. phagocytophilum* growth in tick salivary glands

**Figure 6:**
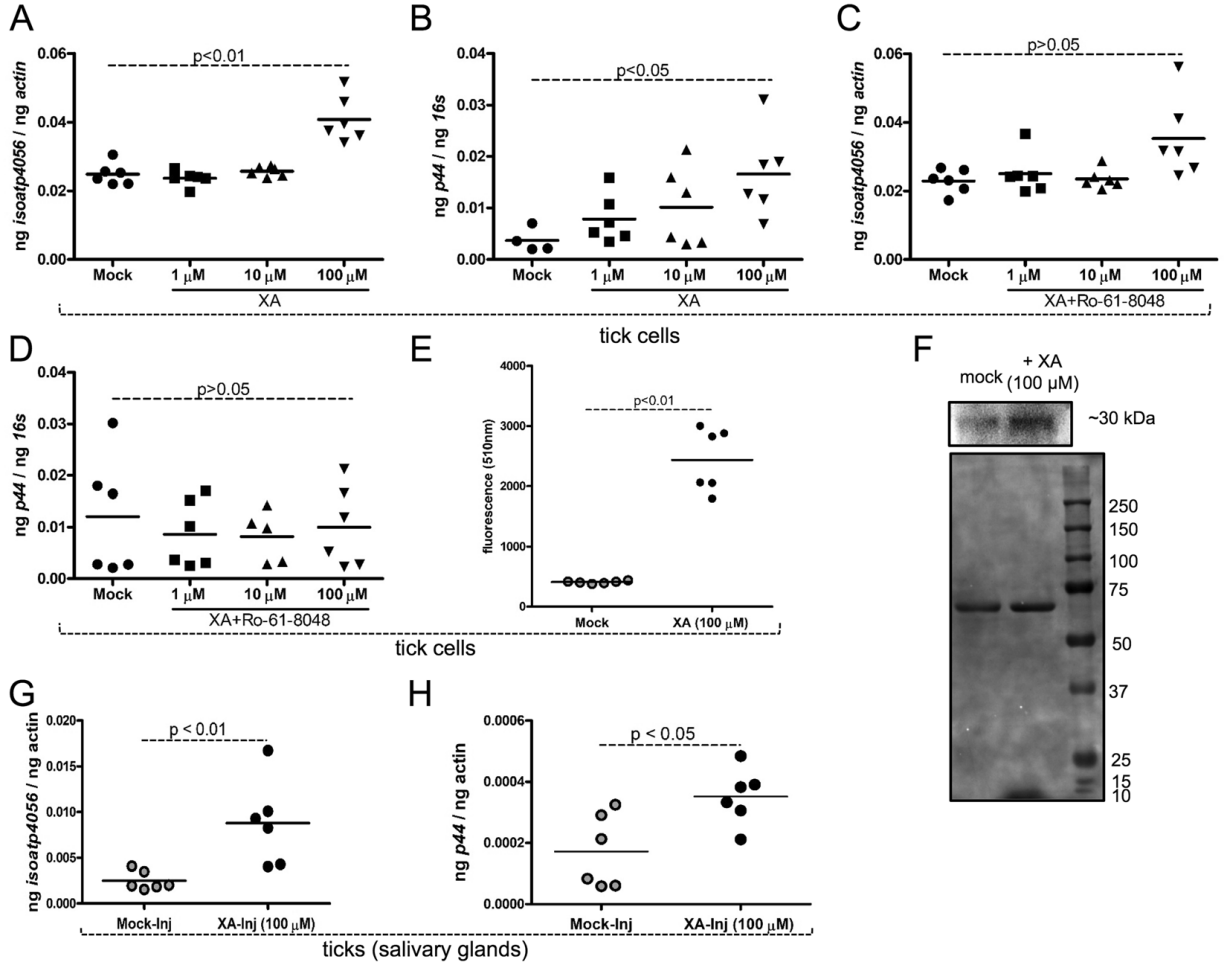
Exogenous treatment with XA induces *isoatp4056* expression and *A. phagocytophilum* burden in tick salivary glands and tick cells. QRT-PCR analysis showing expression of *isoatp4056* **(A** and **C**) and bacterial loads (**B** and **D**) upon treatment with xanthurenic acid (**A** and **B**) or xanthurenic acid plus Ro-61-8048 (an inhibitor of XA biosynthesis) at (**C** and **D**) different doses in *A. phagocytophilum*-infected tick cells is shown. Mock controls were treated with the same amount of solvent used for the preparation of XA and the inhibitor. Each circle/square/triangle/inverted triangle represents data from one independent well of the culture plate performed in duplicates. (**E**) Fluorometer measurements of tick cells infected with GFP-*A. phagocytophilum* treated with mock (gray circles) or 100 μM XA (black circles) at 510 nm is shown. Each circle represents data from one independent well of the culture plate performed in duplicates. (**F**) Immunoblotting analysis with anti-GFP antibody showing levels of GFP protein in tick cells infected with GFP-*A. phagocytophilum* treated with mock or XA (100 μM). Levels of proteins observed on Ponceau stained membrane (used for immunoblotting analysis) serves as a loading control. QRT-PCR analysis showing levels of *isoatp4056* (**G**) or bacterial burden (**H**) after 24 h post-microinjection in mock- or XA-injected *A. phagocytophilum*-infected tick salivary glands is shown. Each circle represents data from pair of salivary glands isolated from individual unfed nymphal ticks. P value from non-paired Student’s t test is shown.

Due to the downregulation of *isoatp4056* mRNA in the *kat*-dsRNA-treated *A. phagocytophilum*-infected tick cells (Fig. 5B), we first tested whether exogenously added XA induces *isoatp4056* expression and bacterial burden in these cells. Tick cells were treated with XA or Ro-61-8048, an inhibitor of XA biosynthesis^37,38^, or both in different doses followed by *A. phagocytophilum* infection as described in the methods section. QRT-PCR analysis revealed that *isoatp4056* levels (Fig. 6A) and *A. phagocytophilum* burden (Fig. 6B) were significantly (P < 0.05) increased in tick cells upon treatment with 100 μM of XA. However, no significant increase (P > 0.05) in the *isoatp4056* levels (Fig. 6C) or *A. phagocytopohilum* burden (Fig. 6D) was evident with the treatment of XA in the presence of inhibitor Ro-61-8048. Treatment of tick cells with Ro-61-8048 alone did not show any effect on *isoatp4056* mRNA levels (Supplementary Fig. 4). To further support these observations, tick cells were treated with 100 μM of XA or mock solutions followed by GFP-*A. phagocytophilum* infection. Tick cells were collected after 48 p.i. and processed for fluorescence microscopy and fluorometer measurements. Tick cells treated with 100 μM of XA showed numerous GFP positive cells in comparison to mock-treated cells (Supplementary Fig. 5). Fluorometer measurements revealed increased fluorescence in tick cells treated with 100 μM of XA in comparison to mock treated control (Fig. 6E and Supplementary Fig. 6). Immunoblotting further supported this observation with increased GFP protein levels in XA-treated tick cells in comparison to the mock-treated controls (Fig. 6F and Supplementary Figure 7A). The total protein profile image after Ponceau S staining served as loading control in the immunoblotting analysis (Fig. 6F).

To test whether XA induces *isoatp4056* expression and bacterial burden in ticks, *A. phagocytophilum*-infected unfed ticks were injected with either 100 μM of XA or mock control. At 24 post-microinjection, QRT-PCR analysis revealed significant (P < 0.05) increase in *isoatp4056* transcripts (Fig. 6G) and bacterial burden (Fig. 6H) in the salivary glands of XA-injected *A. phagocytophilum*-infected ticks in comparison to the infected mock-treated control. Collectively, these results indicate that XA play a role in the regulation of *isoatp4056* expression that is essential for the colonization of *A. phagocytophilum* in tick salivary glands.

### *A. phagocytophilum* and XA influences *isoatp4056* promoter

**Figure 7:**
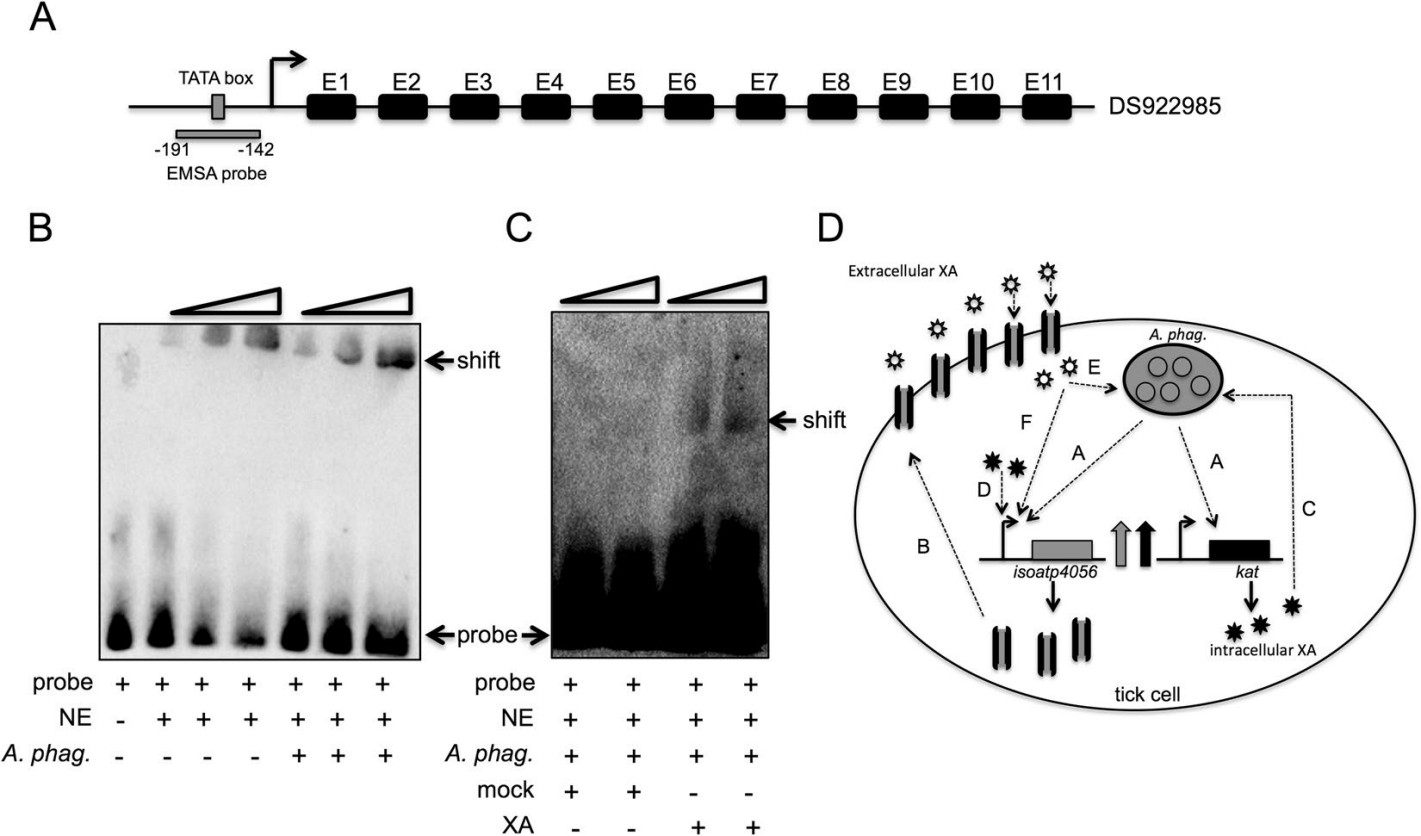
*A. phagocytophilum* and XA promotes increased binding of tick nuclear proteins on the *isoatp4056* promoter. (**A**) Schematic representation (not to scale) of the *isoatp4056* genomic region comprising of 11 exons (indicated as E1-11) and a putative promoter and a TATA-binding region is shown. GenBank accession number for the genomic sequence is provided. The position of −191 and −142 is labeled considering + 1 for the first nucleotide of exon 1 (E1). (**B**) EMSA gel image showing increased shift of *isoatp4056* TATA-binding promoter region in the presence of *A. phagocytophilum*. EMSAs were performed with the biotin-labeled *isoatp4056* promoter TATA-binding regions and uninfected or *A. phagocytophilum*–infected ticks nuclear extract. Wedges indicate increasing amounts of nuclear extracts (1, 2, 4 µg). Probe and shifted bands are labeled. (**C**) EMSAs performed with the biotin-labeled *isoatp4056* promoter TATA-binding region and mock or XA-treated *A. phagocytophilum*–infected tick cell nuclear extracts. Wedges indicate increasing amounts of nuclear extracts (1, 1.5 µg). Gel shifts and the *isoatp4056*-free probes are indicated with arrows. (**D**) Model for the role of IsOATP4056 and KAT in *A. phagocytophilum* survival in tick cells. *A. phagocytophilum* up-regulates *isoatp4056* and *kat* genes upon entry into tick salivary gland cells (**A**). Upregulation of these genes results in the increased production of IsOATP4056 that could be targeted to the plasma membrane as a transporter for intake of extracellular XA shown as open asterisk (**B**). Upregulation of *kat* gene results in increased production of intracellular XA (closed asterisk) that could facilitate *A. phagocytophilum* replication and colonization (**C**) and/or influence *isoatp4056* promoter to make more IsOATP4056 (**D**). Intake of extracellular XA (open asterisk) could also participate in the increase of *A. phagocytophilum* burden (**E**) or regulation of *isoatp4056* promoter (**F**). *A. phagocytophilum* is shown as morulae in the vacuole (gray color). Picture is not drawn to the scale.

As the presence of *A. phagocytophilum* and exogenous addition of XA increased *isoatp4056* mRNA levels, we tested whether the influence of both these factors act at the *isoatp4056* promoter level. We identified a putative *isoatp4056* promoter region in the *I. scapularis* genome from the GenBank sequence DS922985 (Fig. 7A). Electrophoretic mobility shift assay (EMSA) showed increased binding of biotinylated *isoatp4056* DNA promoter probe with proteins in the nuclear extracts prepared from *A. phagocytophilum*-infected nymphal ticks in comparison to the proteins in the extracts prepared from uninfected nymphal ticks (Fig. 7B and Supplementary Fig. 7B), further demonstrating the influence of this bacterium in the regulation of *isoatp4056* gene expression. We did not find any shift when the same probe was incubated with XA alone (without any nuclear extracts) ruling out the possibility of direct binding of XA on the promoter region (Supplementary Fig. 7C). However, we noticed a clear band shift of *isoatp4056* probe when incubated with nuclear extracts prepared from *A. phagocytophilum*-infected tick cells treated with 100 μM XA (Fig. 7C and Supplementary Fig. 7D). No shift was evident when the *isoatp4056* probe was incubated with nuclear extracts prepared from *A. phagocytophilum*-infected tick cells treated with mock (Fig. 7C and Supplementary Fig. 7D). These results further indicate that *A. phagocytophilum* and XA play a role in mediating *isoatp4056* gene expression important for bacterial survival in ticks.

## Discussion

Over the last decade, significant number of studies have focused on understanding the interactions of various pathogens with their vector hosts^10,21,39–44^. Arthropods have different stages in their life cycle^1,2^. Understanding how vector-borne pathogens persists for longer periods and survive at different developmental stages, particularly in unfed stages, in medically important arthropods will provide novel information on the mechanisms of infection and suggest new strategies to combat diseases.

OATPs are highly conserved molecules among various arthropods, including ticks, mosquitoes and lice^35^. So far, no studies have addressed the role of these conserved molecular transporters in vector-pathogen interactions. This key study provides a model to understand the interplay between highly conserved molecular repertoires and pathways in the vector host that are manipulated by pathogens for their survival. Our study elucidates that in the unfed stage of nymphs, where, *A. phagocytophilum* is transstadially maintained, the *isoatp4056* and *kat* genes are upregulated in the salivary glands. Upon upregulation, increased IsOATP4056 production could lead to targeting this protein to the plasma membrane as a transporter for increased uptake of extracellular XA from the extracellular environment, such as from hemolymph (Fig. 7D). Upregulation of *kat* transcripts suggest an increase in the intracellular XA levels that might be essential for *A. phagocytophilum* colonization and/or *isoatp4056* promoter regulation (Fig. 7D). The intake of extracellular XA by IsOATP4056 and increased production of intracellular XA (due to high KAT levels) could be important for *A. phagocytophilum* colonization and survival in the tick salivary glands during unfed stage.

The finding that only one out of nine OATPs was influenced by *A. phagocytophilum* in the tick salivary glands shows the important and specific role for IsOATP4056 in the interactions of this obligate intracellular bacterium with its vector host. Upon acquisition from the murine host, *A. phagocytophilum* enters the gut, exits to the hemocoel, and colonizes in the salivary glands^13^. Our current findings, along with the previously published study that reported significantly higher levels of *isoatp4056* transcripts in the uninfected tick salivary glands in comparison to the midgut tissues^35^ suggest that *A. phagocytophilum* require this specific OATP for its colonization and survival in the salivary glands. The observation of higher levels of *isoatp4056* mRNA in unfed ticks and ticks cells along with RNAi analysis and inhibitor studies support the role for IsOATP4056 in *A. phagocytophilum* colonization and survival in its vector host.

The *isoatp4134* transcripts are exclusively expressed only in the salivary glands of the uninfected tick^35^. However, we did not find any differences in the expression of this gene in the presence of *A. phagocytophilum* (Fig. 1). The other OATPs, *isoatp*0726, *isoatp*2114, *isoatp*2116, *isoatp4548*, *isoatp4550*, *isoatp5126* are reported to be ubiquitously and differentially expressed in various tick tissues^35^. The expression of these OATPs was not affected by the presence of *A. phagocytophilum* (Fig. 1). The upregulation of *isoatp5621* in the presence of *A. phagocytophilum* in whole ticks but not in salivary glands or guts suggests a role for this OATP in other tick tissues involved in inter-organ communication within ticks during infection. Our future studies on *isoatp5621* may reveal undefined roles for this OATP in tick-pathogen interactions.

Our previous studies showed that *A. phagocytophilum* has a unique tropism in the arthropod vector. It serves as a facultative beneficial microbe for tick survival at cold temperatures^16^ and modulates extended actin phosphorylation to regulate arthropod gene expression^19^. This study shows that it also causes interplay between organic anion transporters and XA during vector-pathogen interactions. XA was shown to induce gametogenesis of *P. falciparum* in mosquitoes^45–48^. However, the molecular mechanism for its transport and interactions with the malaria parasite has not been explored. Our study suggests that IsOATP4056 may act as a novel transporter for XA and elucidates new information on the role for XA in the regulation of gene expression. The EMSA data (Fig. 7) suggests that XA may act as a co-factor for some of the transcription factors that could act as activators of gene expression; for example, the transcription factors that bind to the *isoatp*4056 promoter. The reduced levels of *isoatp4056* mRNA in *kat*-dsRNA-treated tick cells and increased levels of *isoatp4056* expression upon exogenous addition of XA further support this hypothesis.

XA is present in vertebrate blood and urine at concentrations of 0.7 and 5–10 μM, respectively^49,50^. As larval ticks feed on a vertebrate animal, it may acquire host XA and retain it through different developmental stages, including the unfed nymphal stage. Therefore, the role of exogenous XA from the vertebrate host in the colonization of *A. phagocytophilum* in unfed ticks cannot be ruled out. Studies on these aspects would unravel several interesting aspects of *A. phagocytophilum* persistence and colonization in these ticks.

In summary, this study not only provides novel information on the modulation of the interplay between OATPs and the tryptophan metabolite, XA, by *A. phagocytophilum* for its survival in the vector host but also lead to development of better strategies to interrupt the survival of this bacterium and perhaps other vector-borne rickettsial pathogens of medical importance.

## Methods

### Bacterial isolates, Ticks and tick cell line

*A. phagocytophilum* isolate NCH-1 was used in the studies that involved *in vivo* work with ticks and mice. Isolate HZ and HGE1-GFP isolates were used in the *in vitro* cell line experiments. Wherever necessary, NCH-1 and HZ isolates are referred as *A. phagocytophilum*, and HGE1-GFP is referred as GFP-*A. phagocytophilum*. *Escherichia coli* DH5alpha strain was used as a cloning host for generating different plasmids. The laboratory-reared specimens of *I. scapularis* ticks were used throughout the study. Ticks used in this study are larvae and nymphs obtained from a continuously maintained tick colony at the Department of Epidemiology and Public Health, Yale University (New Haven, CT) and/or Department of Entomology, Connecticut Agricultural Experiment Station (New Haven, CT). The *I. scapularis* tick cell line ISE6 and HGE1-GFP *A. phagocytophilum* strain was a gift from Dr. Ulrike Munderloh at the University of Minnesota (St Paul, MN) and was maintained as described^26^. Isolate HZ-1 was a gift from Dr. Joao Pedra at the University of Maryland (Baltimore, MD) and was maintained as described^51^.

### Mice

C3H/HeN (female mice, 4–6 weeks, CharlesRiver Laboratories, USA) mice were used throughout this study. To generate uninfected or *A. phagocytophilum*–infected unfed nymphs, larvae were fed on either uninfected or *A. phagocytophilum*–infected C3H/HeN mice and allowed to molt. Tick rearing was conducted in an incubator at 23 ± 2 °C with 95% relative humidity and a 14/10 hour light/dark photoperiod regiment. In acquisition experiments, dsRNA- or mock-treated uninfected nymphs were fed on *A. phagocytophilum*-infected mice and upon repletion were processed further for RNA and DNA extractions. Animal husbandry was provided under the Association for Assessment and Accreditation of Laboratory Animal Care Program at the current institution. Acepromazine tranquilizer was administered to the animals prior to handling to minimize anxiety and/or discomfort and all efforts were made to minimize suffering.

### Ethics Statement

All animal work in this study was carried out in strict accordance with the recommendations in the Guide for the Care and Use of Laboratory Animals of the National Institute of Health. The protocol used in this study (permit number: 16–017) was approved by the Old Dominion University Institutional Animal Care and Use Committee (Animal Welfare Assurance Number: A3172-01).

### RNA or DNA extractions and Quantitative Real-time PCR (QRT-PCR) Analysis

Total RNA from unfed or fed ticks was generated using the Aurum Total RNA mini kit (Bio-Rad, USA) following the manufacturer’s instructions. RNA was converted to cDNA using BioRAD cDNA synthesis kit (BioRAD, USA). The generated cDNA was used as a template for quantifying nine OATPs or KAT transcripts using the oligonucleotides mentioned in Table S1. As an internal control and to normalize the amount of template, *I*. *scapularis beta-actin* was quantified using oligonucleotides as mentioned in Table S1. QRT-PCR was performed as described^52^ using CFX96 QPCR machine (BioRad, USA) and iQ-SYBR Green Supermix (BioRad, USA). To quantify bacterial burden in ticks, genomic DNA from *A*. *phagocytophilum*–infected unfed or fed nymphs or tick cells was extracted using DNeasy kit (QIAGEN) and processed for PCR with primers specific for the *A. phagocytophilum p44* gene as shown in Supplementary Table 2. In QRT-PCR reactions, the standard curve was generated using 10-fold serial dilutions starting from 1 ng to 0.00001 ng of known quantities of respective fragments.

### dsRNA synthesis and tick microinjections

The dsRNA synthesis was performed as described^16,19^. Briefly, the *isoatp4056- or kat-*dsRNA fragments were generated by PCR using gene specific primers containing BglII (forward primer) and KpnI (reverse primer) restriction enzyme sites using oligonucleotides mentioned in Table S1. The fragments containing *isoatp4056* or *kat* sequences were purified and cloned into BglII-KpnI sites of the L4440 double T7 Script II vector. The dsRNA’s complementary to *isoatp4056* or *kat* gene sequences were synthesized using the MEGAscript RNAi Kit (Ambion Inc.) following manufacturer’s instructions. Microinjections of mock (buffer alone) and *isoatp4056-*dsRNAs were performed as described^16,19^. Briefly, 1 μg of L4440 vector containing *isoatp4056* sequences was used as a template to prepare dsRNA. The prepared dsRNA was eluted in 50 μl of elution buffer (Ambion Inc.). Microinjections were performed (~4.2 nl/tick, 1 × 10^12^ molecules/μl) into the bodies of uninfected unfed nymphs. Microinjected ticks were incubated at room temperature for 2–3 hours for acclimatization in a desiccator and fed on *A. phagocytophilum*–infected or naïve mice. Repleted ticks were then analyzed for *isoatp4056* expression to determine silencing efficiency by QRT-PCR using oligonucleotides as mentioned in the Table S1.

Microinjections of XA into ticks were performed in a similar way. Briefly, *A. phagocytophilum*-infected unfed ticks were injected with 100 μM XA or mock solution (~4.2 nl/tick) into the body. Injected ticks were incubated for 24 h and then processed for salivary gland isolation followed by QRT-PCR analysis to evaluate *isoatp4056* expression and *A. phagocytophilum* burden.

### Tick cell line experiments with xanthurenic acid (XA) and the inhibitors

Strain *A. phagocytophilum* HZ or GFP-*A. phagocytophilum* were maintained in Human promyelocytic cell line (HL-60, American Type Culture Collection, USA), and cell-free bacteria isolated from these cells were used for *in vitro* infection studies as described^53^. Both XA and Ro-61-8048, an inhibitor of XA biosynthesis^38^, were purchased from Sigma (USA). OATP inhibitor (±-sulfinpyrazone) was purchased from Santa Cruz Biotechnology Inc. Stocks (10 mM) of XA or Ro-61-8048 or ± -sulfinpyrazone were made in 0.5 N NaOH solutions. A 1:10 dilution of the stocks were prepared in 1x PBS to a final concentration of 1 mM and used for all experiments. Mock solution was prepared in a similar way but without XA or Ro-61-8048 or ± -sulfinpyrazone. Tick cells (1 × 10^5^) were seeded onto 12 well plates and incubated for 16–20 hours. Following incubation, tick cells were treated with different concentrations of XA or Ro-61-8048 (1, 10, 100 μM) or both or 100 μM of ± -sulfinpyrazone for 4 hours followed by *A. phagocytophilum* infection. Equal volumes of mock solution (corresponding to 100 μM volume) were added to control cells. The cells were then incubated for 48 h (for XA or Ro-61-8048 treatment experiments) or 24 h (for ± -sulfinpyrazone treatment experiments) and processed further for RNA or DNA extractions to measure OATP transcripts and *A. phagocytophilum* loads.

### Tick cell line silencing experiments

For silencing *isoatp4056* or *kat* expression in ISE6 cells, Lipofectamine transfection reagent (ThermoFisher Scientific/Invitrogen) was used. Briefly, 5 × 10^5^ tick cells were seeded in L-15B300 medium on to 12 well plates and incubated for 24 hours. At 24 h, 500 ng of dsRNA mixed with Lipofectamine reagent was added. After 6 hours of addition of dsRNA and Lipofectamine, 2x L15-B300 medium was added and plates were further incubated for additional 16 h. Cell-free *A. phagocytophilum* (isolated from infected HL-60 cells) was added after 24 h post transfection and cells were incubated further for 24 h and processed for RNA or DNA extractions. Representative images (collected with EVOS FL microscope) at different time points for mock or *isoatp4056- or kat-*dsRNA-treated tick cells are shown in Supplementary Figures 2 and 3. Silencing efficiency and the bacterial burden was determined as mentioned in other sections.

### Spectrophotometry, fluorescence microscopy and immunoblotting

Tick cells (1 × 10^5^) were seeded onto 12 well plates (containing glass coverslips at the bottom in each well) and incubated for 16–20 hours. XA treatment and infection with GFP-*A. phagocytophilum* procedures were followed as described in the *in vitro* cell line experiment section. After 48 h of incubation, coverslips were removed and placed in empty 12 well plate. Cells were immediately fixed with 4% paraformaldehyde at 37 °C for 15 min. Images were acquired using EVOS cell imaging system (Invitrogen/Thermo Fisher Scientific, USA). For Fluorescence spectrophotometry, cells infected with GFP-*A. phagocytophilum* were collected after 48 h post XA treatment, centrifuged and the cell pellet was re-suspended in 1x PBS. Fluorescence was measured with a Fluorimeter (Tecan, USA) with excitation at 384 nm and emission at 510 nm. The spectrum generated in this wavelength range is shown in the Supplementary Figure 6. Immunoblotting was performed as described^19,54,55^. Total lysates from ISE6 tick cells were prepared in modified RIPA buffer (BioExpress/VWR, USA) supplemented with EDTA-free protease inhibitor cocktail (Sigma, USA). Protein concentrations were determined by BCA protein assay kit (Pierce/ThermoScientific, USA). Primary and secondary antibodies were obtained from Santa Cruz Biotechnology Inc. (USA). Thirty micrograms of total lysates from each group were loaded onto a 12% reducing stain-free SDS-PAGE gels (NuPAGE) for immunoblotting.

### Preparation of nuclear extracts and electrophoretic mobility shift assay (EMSA)

Nuclear extracts from uninfected or *A. phagocytophilum*-infected ticks or tick cells were prepared using NE-PER nuclear and cytoplasmic extraction kit (Pierce/Thermo Fisher Scientific, USA) according to manufacturer instructions. EMSA assays were performed as described^19^. Briefly, complimentary oligonucleotides consisting of *isoatp4056* putative promoter TATA- binding region were annealed and biotin labeled according to Pierce Biotin 3’ End DNA labeling kit (Pierce/Thermo Fisher Scientific, USA). The labeled oligonucleotides were added to a 20-μl reaction mix consisting of reagents from LightShift EMSA optimization and control kit (Pierce/Thermo Fisher Scientific, USA) and nuclear extracts prepared from either nymphal ticks or ISE6 tick cells. In some cases, nuclear lysates prepared from tick cells treated with 100 μM XA or mock (corresponding to equal volume of 100 μM XA) were used. The entire reactions were incubated at room temperature for 25 min and loaded onto 6% native DNA polyacrylamide gel. The gel was initially pre-run at 100 V with 0.5% Tris-Borate-EDTA and again run with samples at the same settings and conditions. Later, the gel was transferred and processed following Chemiluminescent nucleic acid detection module recommendations (Pierce/Thermo Fisher Scientific, USA). Images were captured using ChemiDoc MP imager (BioRad, USA).

### Salivary gland and gut isolation

Pairs of salivary glands or whole gut from individual unfed nymphal ticks were dissected in sterile 1x phosphate buffer saline and processed for homogenization in Lysis buffer (Aurum Total RNA kit, Bio-Rad) for RNA extractions following manufacturer’s recommendations. The extracted RNA samples were later converted to cDNA and used for QRT-PCR analysis.

### GenBank Accession numbers for *I. scapularis* OATPs and KAT

The following are the GenBank accession numbers for the nine OATPs and *kat* genes: XM_002400726 (*isoatp0726*), XM_002412114 (*isoatp2114*), XM_002412116 (*isoatp2116*), XM_002414056 (*isoatp*4056), XM_002434134 (*isoatp*4134), XM_002404548 (*isoatp*4548), XM_002404550 (*isoatp*4550), XM_002415126 (*isoatp*5126), XM_002435621 (*isoatp*5621) and *kat* (XM_002401267).

### Statistics

Statistical significance in the data sets was analyzed using GraphPad Prism6 software and Microsoft Excel 2010. For data to compare two means, the non-paired Student t-test was performed. P values of < 0.05 were considered significant in all analysis. Wherever necessary, statistical test and P values used are reported.

## Electronic supplementary material

Supplementary information
