# Supplementary material for: Human rickettsial pathogen modulates arthropod organic anion transporting polypeptide and tryptophan pathway for its survival in ticks
Source: Sci Rep. 2017 Oct 16;7:13256. doi: 10.1038/s41598-017-13559-x (PMC5643405; doi:10.1038/s41598-017-13559-x)
Supplement: Supplementary file 1 — Supplementary information [file 41598_2017_13559_MOESM1_ESM.pdf]

Human rickettsial pathogen modulates arthropod organic anion transporting polypeptide and tryptophan pathway for its survival in ticks

Vikas Taank [1], Shovan Dutta [1], Amrita Dasgupta [1, #], Tanner K Steeves [2], Durland Fish [2], John F Anderson [3], Hameeda Sultana [1, 4], and Girish Neelakanta [1, 4, $]

Supplementary information

Supplementary Figures

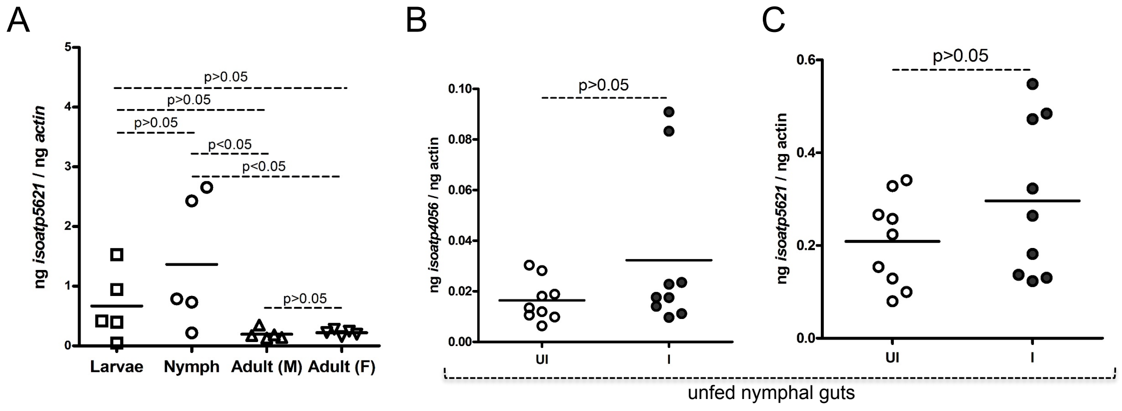

Supplementary Figure 1

Supplementary Figure 1: *isoatp5621* expression at different developmental stages of ticks. A) QRT-PCR analysis showing expression of *isoatp5621* at different tick developmental stages in uninfected unfed ticks. Each square/circle/triangle/inverted triangle represents one tick. M indicates adult males and F indicates adult female ticks. Data for larvae samples was obtained from 5-7 pooled ticks. QRT-PCR analysis showing expression of *isoatp4056* (B) or *isoatp5621* (C) in unfed uninfected or *A. phagocytophilum*-infected nymphal guts is shown. Each circle represents one gut sample isolated from an individual tick. The levels of *isoatp4056 or isoatp5621* mRNA was normalized to tick beta-actin. P value from non-paired Student's t-test is shown.

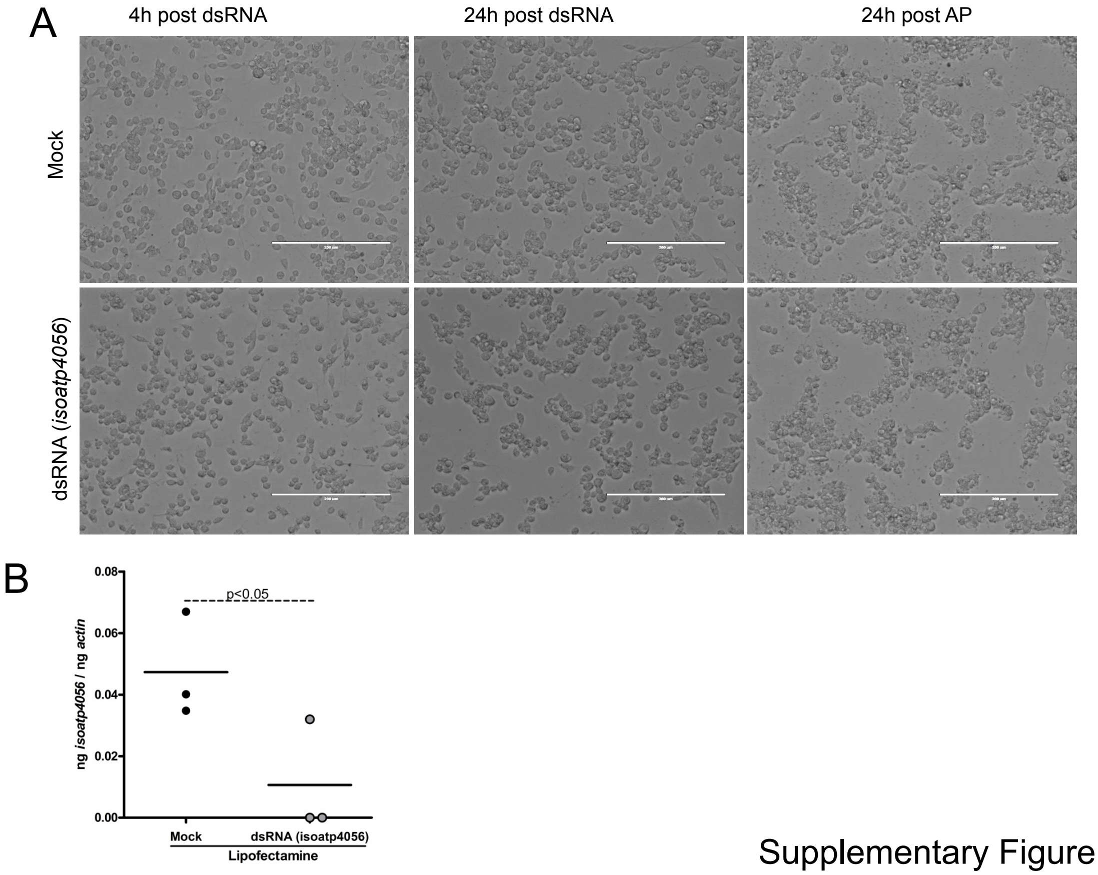

Supplementary Figure 2

Supplementary Figure 2: *isoatp4056*-dsRNA treatment showed no morphological changes in tick cells. A) Representative images (3 images for each group) of *A. phagocytophilum*-infected mock treated or *A. phagocytophilum*-infected *isoatp4056*-dsRNA treated at 4 h and 24 h before infection and 24 h post-infection (last panel) is shown. Scale indicates 200 µm. B) QRT-PCR analysis showing expression of *isoatp4056* mRNA in mock or *isoatp4056*-dsRNA-treated uninfected tick cells. Levels of *isoatp4056* were normalized to tick beta-actin levels. P value from non-paired Student's t-test is shown.

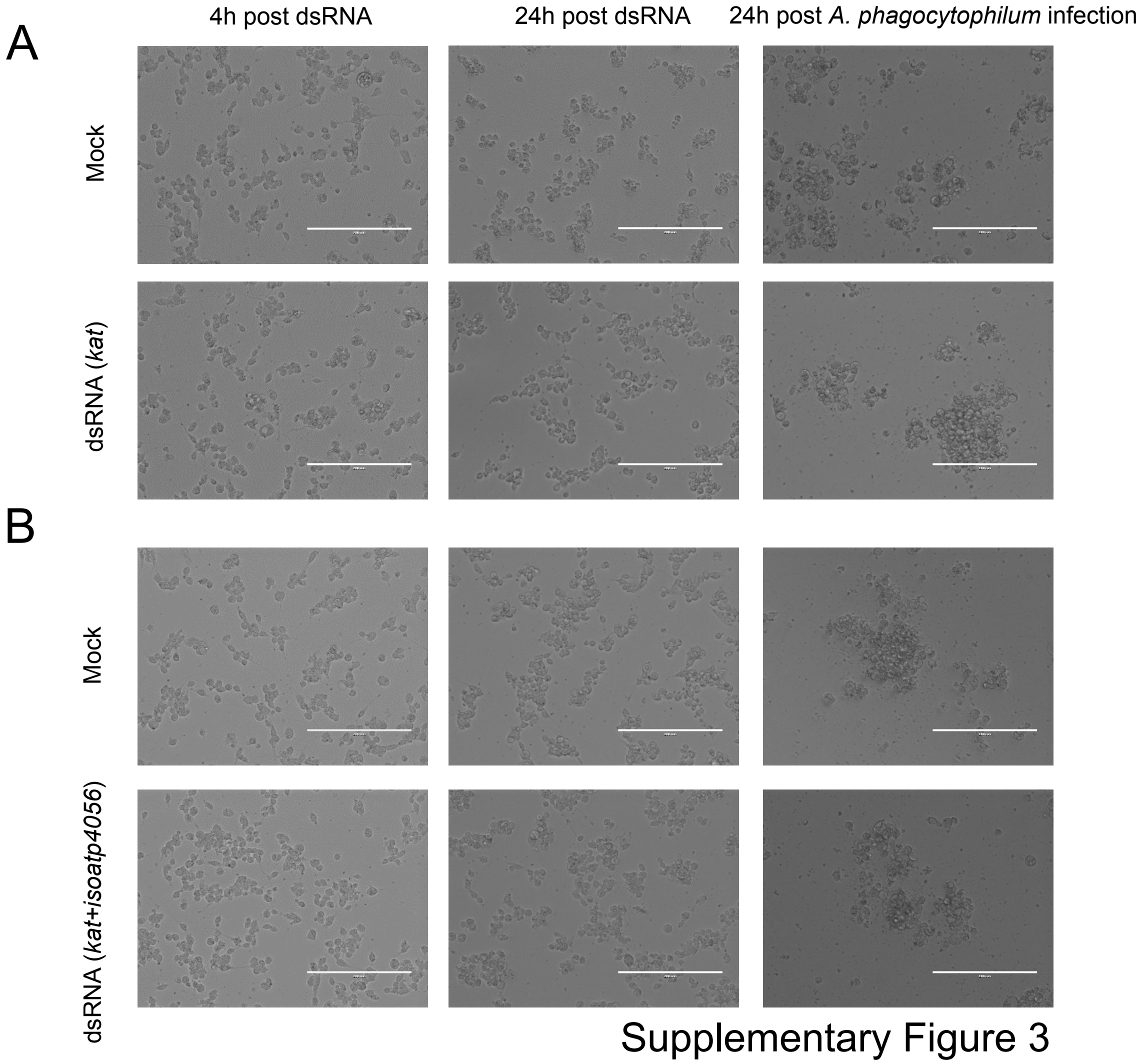

Supplementary Figure 3: *kat*- or *kat+isoatp4056*-dsRNA treatment showed no morphological changes in tick cells. Representative images (3 images for each group) of *A. phagocytophilum*-infected mock treated or *A. phagocytophilum*-infected *kat-* (A) *or kat+isoatp4056*-dsRNA (B) treated at 4 h and 24 h before infection and 24 h post-infection (last panel) is shown. Scale indicates 200 µm.

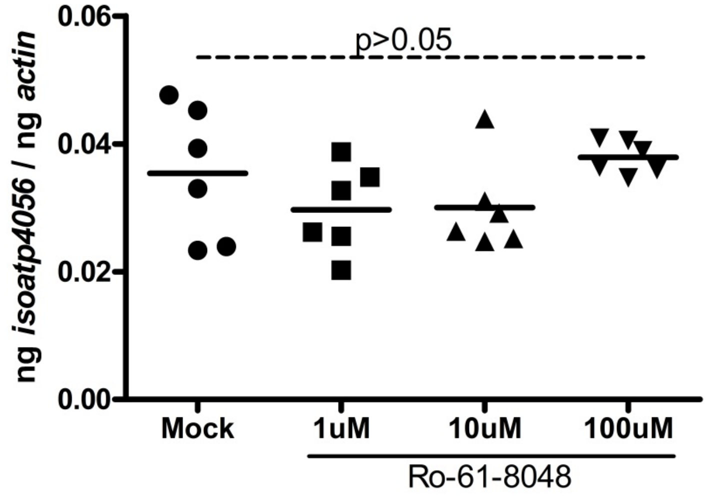

Supplementary Figure 4

Supplementary Figure 4: Exogenous treatment with Ro-61-8048 had no effect on *isoatp4056* expression in tick cells. QRT-PCR analysis showing expression of *isoatp4056* upon treatment with Ro-61-8048 (an inhibitor of XA biosynthesis) at different doses in *A. phagocytophilum*-infected tick cells is shown. Mock controls were treated with the same amount of solvent used for the preparation of the inhibitor. Each circle/square/triangle/inverted triangle represents data from one independent well of the culture plate performed in duplicates. P value from Student's t-test is shown.

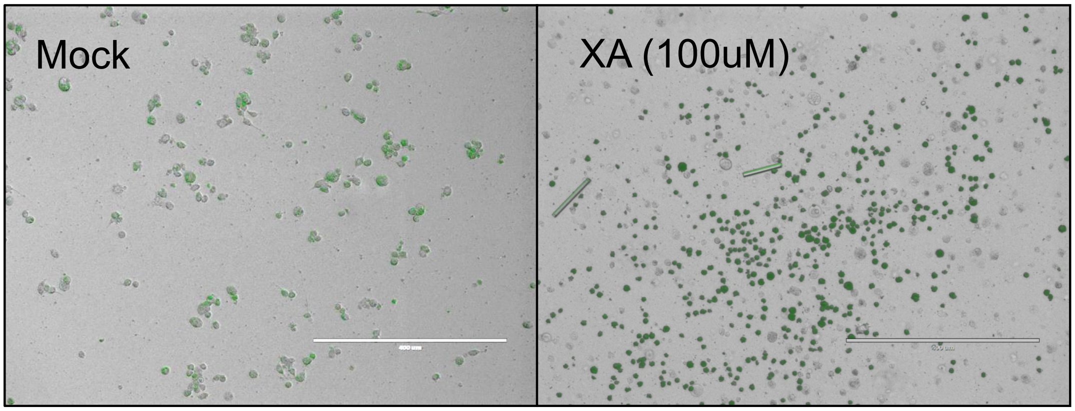

Supplementary Figure 5

Supplementary Figure 5: Exogenous treatment with XA increases GFP-*A. phagocytophilum* growth in tick cells. Representative images of tick cells infected with GFP-*A. phagocytophilum* treated with mock or XA (100 µM) is shown. Scale bar represents 400 µm. GFP fluorescence is evident as green color in the image.

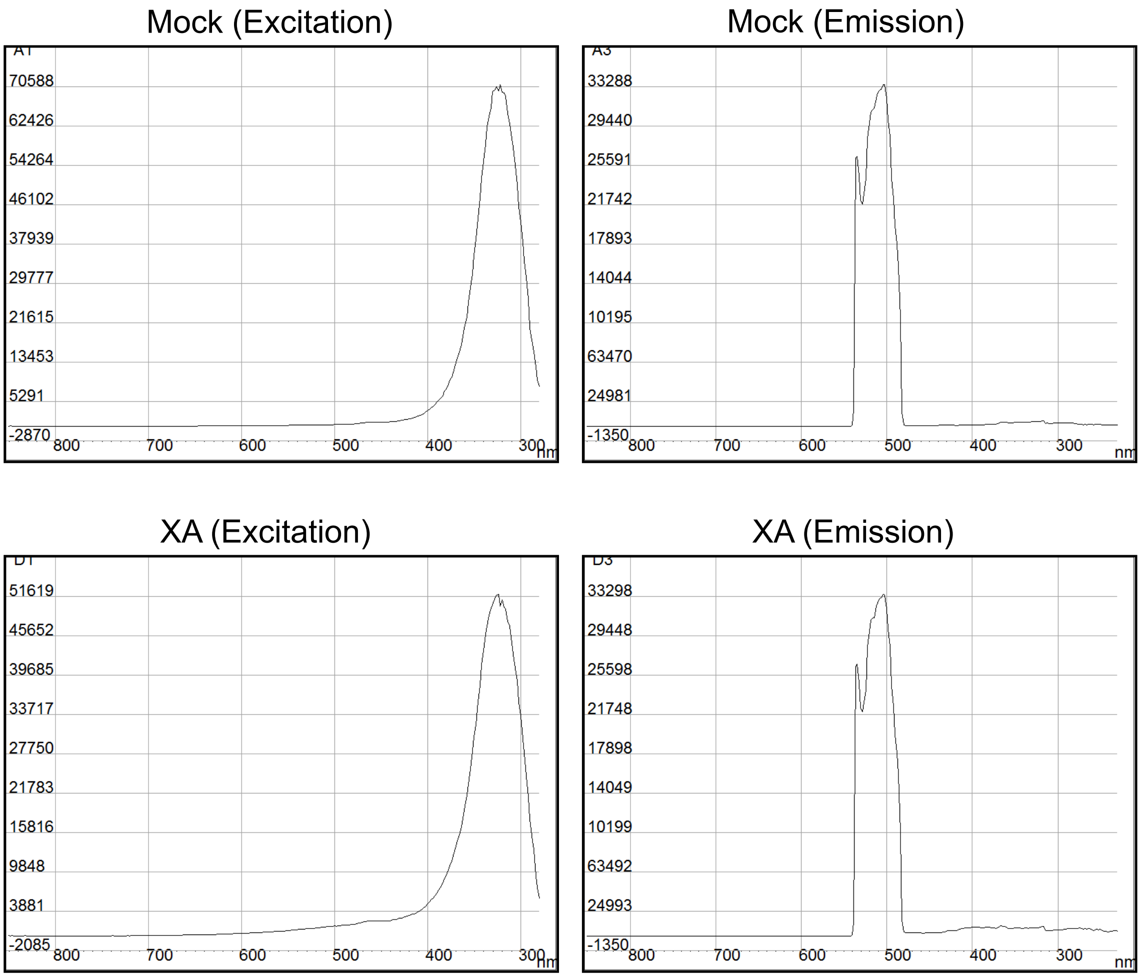

Supplementary Figure 6

Supplementary Figure 6: Fluorometer spectrum data for GFP-*A. phagocytophilum*-infected tick cells treated with XA. A) Representative spectrum data from fluorometer for tick cells infected with GFP-*A. phagocytophilum* treated with mock or XA (100 µM) is shown. Both excitation (384 nm) and emission (510 nm) spectrum for mock or XA treatment are shown.

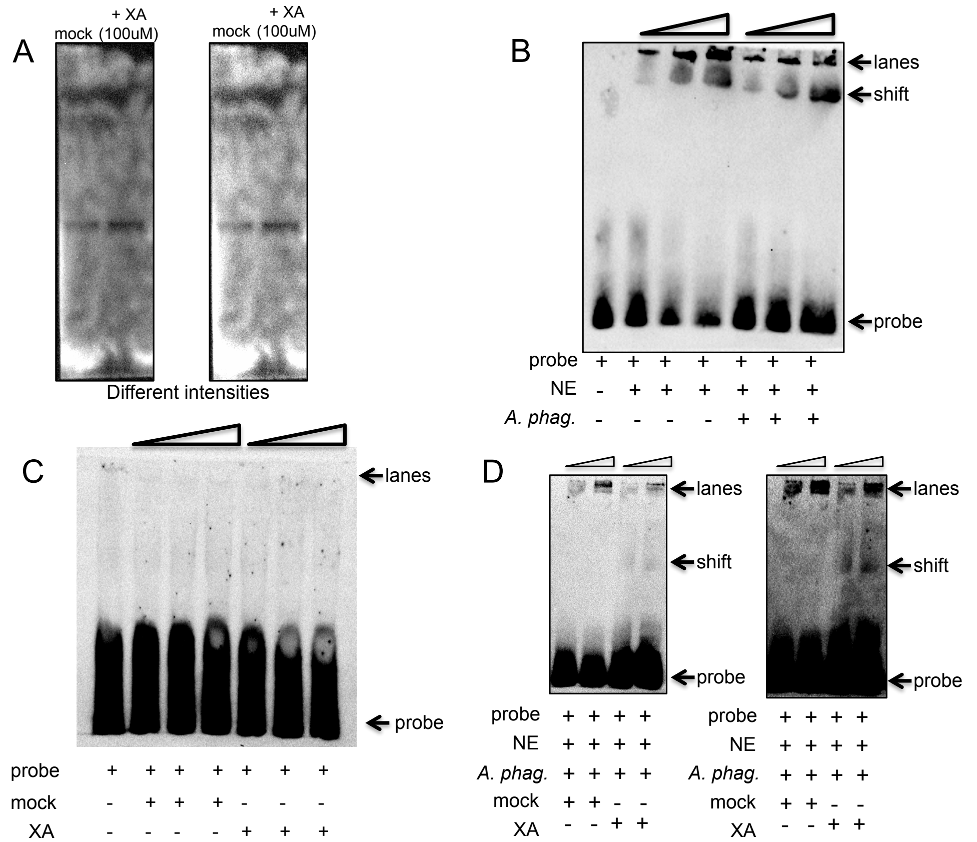

Supplementary Figure 7

Supplementary Figure 7: Exogenous addition of XA alone does not affect *isoatp4056* promoter. A) Immunoblotting analysis with anti-GFP antibody showing levels of GFP protein in tick cells infected with GFP-*A. phagocytophilum* treated with mock or XA (100 µM). Whole blot (in different intensities) for the image in Figure 6F is shown. B) EMSA gel image showing increased shift of *isoatp4056* TATA-binding promoter region in the presence of *A. phagocytophilum*. EMSAs were performed with the biotin-labeled *isoatp4056* promoter TATA-binding regions and uninfected or *A. phagocytophilum*–infected ticks nuclear extract. Wedges indicate increasing amounts of nuclear extracts (1, 2, 4 µg). Whole gel image for the image in Figure 7B is shown. C) EMSAs were performed with the biotin-labeled *isoatp4056* promoter TATA-binding region in the presence of mock or XA. *isoatp4056* probes are indicated with arrows. Wedges indicate increasing amount of mock or XA (1, 10, 100 µM). D) EMSAs performed with the biotin-labeled *isoatp4056* promoter TATA-binding region and mock or XA-treated *A. phagocytophilum*–infected tick cell nuclear extracts. Wedges indicate increasing amounts of nuclear extracts (1, 1.5 µg). Gel shifts and the *isoatp4056*-free probes are indicated with arrows. Whole gel image (in different intensities) for the image in Figure 7C is shown. In all EMSA gel images, probe, shifted bands and lanes are labeled.

Supplementary Table 1: GenBank accession numbers for *I. scapularis* putative genes involved in tryptophan metabolism

Enzyme Number	Enzyme Name	Accession Number	
		VectorBase	GenBank
EC:1.13.11.11	tryptophan 2,3-dioxygenase	ISCW024183	XM_002408230
EC 1.13.11.52	indoleamine 2,3-dioxygenase	Not Found	Not Found
EC:3.5.1.9	arylformamidase	ISCW007881	XM_002408230
EC:1.14.13.9	kynurenine 3-monooxygenase	ISCW011595	XM_002411632
EC 2.6.1.7	kynurenine aminotransferase	ISCW012663	XM_002401267

Supplementary Table 2: Oligonucleotides used in this study.

Primer (5'-3')	Gene, purpose
GGTATCGTGCTCGACTC	tick actin, qpcr
CAGGGCGACGTAGCAG	tick actin, qpcr
GCCACCCCCGCTTAGTGA	tick KAT, qpcr
CGAGATGCTCTCCCCAGTTTTCT	tick KAT, qpcr
CCAGCGTTTAGCAAGATAAGAG	*Anaplasma*, qpcr
GCCCAGTAACAACATCATAAGC	*Anaplasma*, qpcr
TAAACAATTAAAAGCTTTCTT	tick 16S, qpcr
AATCGCTAAAAACGGAACTTA	tick 16S, qpcr
GGGGCGACGGCTGTGT	tick, *isoatp0726*, qpcr
GGGGACAGGTTGGAGGTTTCA	tick, *isoatp0726*, qpcr
GCCCAACTTACATCCTGTCCA	tick, *isoatp2114*, qpcr
CTGCACGGAGCCACAACGA	tick, *isoatp2114*, qpcr
GCGATGGGCCGTTTGTG	tick, *isoatp2116*, qpcr
GCTTCCATATGCGGATGATGA	tick, *isoatp2116*, qpcr
CCGTCACGAAAACGCCTTCA	tick, *isoatp4056*, qpcr
GCTTCCACACGTCCACCTTCT	tick, *isoatp4056*, qpcr
CTCTTGGAAACATCGCCGTG	tick, *isoatp4134*, qpcr
GCGATGACAGTTGCCACGA	tick, *isoatp4134*, qpcr
CATCATCTGCTCGCTAATCCCAC	tick, *isoatp4548*, qpcr
GGGCGGTTGCTTTGAGATAG	tick, *isoatp4548*, qpcr
GGACGAGAACACGCCGACA	tick, *isoatp4550*, qpcr
CGGGGAGCGCTGTCACA	tick, *isoatp4550*, qpcr
GTGTCCGCCAGCTCCATCCT	tick, *isoatp5126*, qpcr
AGATGACGAACGGCAGAGAGGT	tick, *isoatp5126*, qpcr
GTTCATCGGAATCCTTGTGGTAGT	tick, *isoatp5621*, qpcr
GTGGCAGCTTGACACAAGAAGAGT	tick, *isoatp5621*, qpcr
TGAGATCTCGCCCCTGTTGCCTGAAG	tick, *isoatp4056*, RNAi
CGGGTACCAGCACGAGGAAGATACCACA	tick, *isoatp4056*, RNAi
TGAGATCTCAAGACAGCGTCTGGGTGGAGT	tick, *kat*, RNAi
CGGGTACCGTTGTGTGGAGTGTGGTGGCA	tick, *kat*, RNAi
GCCTCGAGCTACGGCGCGAGTGTATACAAAACGACTTTGTCCTCTTGTGC	tick, *isoatp4056*, EMSA probe
GCACAAGAGGACAAAGTCGTTTTGTATACACTCGCGCCGTAGCTCGAGGC	tick, *isoatp4056*, EMSA probe
